# Intranasal Delivery of a Ghrelin Mimetic Engages the Brain Ghrelin Signaling System in Mice

**DOI:** 10.1210/endocr/bqae166

**Published:** 2025-01-15

**Authors:** Renée Poelman, Marie V Le May, Erik Schéle, Iris Stoltenborg, Suzanne L Dickson

**Affiliations:** Department of Physiology/Endocrine, Institute of Neuroscience and Physiology, The Sahlgrenska Academy at the University of Gothenburg, SE-413 90 Gothenburg, Sweden; Department of Physiology/Endocrine, Institute of Neuroscience and Physiology, The Sahlgrenska Academy at the University of Gothenburg, SE-413 90 Gothenburg, Sweden; Department of Physiology/Endocrine, Institute of Neuroscience and Physiology, The Sahlgrenska Academy at the University of Gothenburg, SE-413 90 Gothenburg, Sweden; Department of Physiology/Endocrine, Institute of Neuroscience and Physiology, The Sahlgrenska Academy at the University of Gothenburg, SE-413 90 Gothenburg, Sweden; Department of Physiology/Endocrine, Institute of Neuroscience and Physiology, The Sahlgrenska Academy at the University of Gothenburg, SE-413 90 Gothenburg, Sweden

**Keywords:** ghrelin receptor agonist, GHSR, intranasal, food intake, arcuate nucleus, growth hormone

## Abstract

Ghrelin, the endogenous ligand of the growth hormone secretagogue receptor (GHSR), promotes food intake and other feeding behaviors, and stimulates growth hormone (GH) release from the pituitary. Growth hormone secretagogues (GHS), such as GHRP-6 and MK-0677, are synthetic GHSR ligands that activate orexigenic neuropeptide Y neurons that coexpress agouti-related peptide (AgRP) in the arcuate nucleus of the hypothalamus when administered systemically. Systemic GHRP-6 also stimulates GH release in humans and rats. Thus, GHS and ghrelin have therapeutic relevance in patients who could benefit from its orexigenic and/or GH-releasing effects. This study examined whether intranasal delivery of ghrelin, GHRP-6, or MK-0677 engages the brain ghrelin signaling system.

Effective compounds and doses were selected based on increased food intake after intranasal application in mice. Only GHRP-6 (5 mg/kg) increased food intake without adverse effects, prompting detailed analysis of meal patterns, neuronal activation in the arcuate nucleus (via Fos mapping) and neurochemical identification of *c-fos* messenger RNA (mRNA)-expressing neurons using RNAscope. We also assessed the effect of intranasal GHRP-6 on serum GH levels.

Intranasal GHRP-6 increased food intake by increasing meal frequency and size. Fos expression in the arcuate nucleus was higher in GHRP-6–treated mice than in saline controls. When examining the neurochemical identity of *c-fos*-mRNA–expressing neurons, we found coexpression with 63.5 ± 1.9% *Ghsr* mRNA, 79 ± 6.8% *Agrp* mRNA, and 11.4 ± 2.5% *Ghrh* mRNA, demonstrating GHRP-6's ability to engage arcuate nucleus neurons involved in food intake and GH release. Additionally, intranasal GHRP-6 elevated GH serum levels. These findings suggest that intranasal GHRP-6, but not ghrelin or MK-0677, can engage the brain ghrelin signaling system.

The brain ghrelin signaling system comprises neuronal networks that are involved in orexigenic feeding behaviors and pituitary growth hormone (GH) release. Ghrelin, first isolated in 1999 (1), is a stomach-derived circulating hormone (2) whose most cited physiological role is to increase food intake by stimulating brain pathways involved in hunger and food reward processing. It was identified as the endogenous ligand for the growth hormone secretagogue receptor (GHSR), cloned in 1996 (3). Growth hormone secretagogues (GHSs), which are GHSR agonists and now recognized as ghrelin mimetics, are synthetic ligands that, as their name suggests, powerfully stimulate the release of GH from the pituitary (4). Synthetic GHSR agonists include both peptide (eg, GH-releasing peptide-6, GHRP-6) and nonpeptide (eg, MK-0677) (4, 5) compounds, with the potential to stimulate/rejuvenate the GH axis (6) or that could be used as a provocative test for GH deficiency (7, 8).

GHSs potently increase GH release in rats (4) and in humans (9). In humans, they elevate basal GH release as well as pulse amplitude (10, 11) and amplify growth hormone-releasing hormone (GHRH)-induced GH secretion (9). Pituitary somatotrophs, in addition to expressing receptors for GHRH (12), express GHSR, evidencing a direct pituitary site of action (13). However, GHSs also act via a hypothalamic mechanism, increasing neuronal activation in the arcuate nucleus (shown both electrophysiologically and by Fos protein expression) (14), a subpopulation of neurons that includes GHRH neurons (14-16). It emerged that GHRH neurons were not the only activated population, however; as many as 50% expressed neuropeptide Y (NPY) messenger RNA (mRNA) (15), a population known to coexpress agouti-related peptide (AgRP). These NPY/AgRP neurons are orexigenic (17-19) and have since been identified as an important population for the effects of GHS/ghrelin on food intake and food-linked behaviors (20). Indeed, the AgRP neurons have gained status as “hunger neurons” conveying the negative valence associated with hunger (21). Furthermore, ghrelin's status as a “hunger hormone” that determines when we eat is evidenced not only by its powerful orexigenic effects both in humans and rodents (22-24), but also by studies in humans showing that circulating ghrelin levels increase before mealtimes (25) and in association with hunger (26). As reviewed (27), the central ghrelin system is important not only for consummatory behavior but also for preprandial appetitive behaviors (such as food anticipation) (28) and food reward/motivation (29-33).

In the present study, we sought to determine whether the brain ghrelin signaling system is activated by intranasal delivery of ghrelin and/or by peptide and nonpeptide GHSs, by reproducing some of the aforementioned known effects of systemic delivery of these agonists. There is very little published on intranasal treatment with ghrelin or GHSs. Intranasal administration of the peptide GHS hexarelin and GHRP-6 have both been shown to induce GH release in humans when administered at a dose 30 and 20 times higher than intravenous dose, respectively (34, 35), and intranasal hexarelin accelerates growth in prepubertal children of short stature (36). However, the ability of these compounds to induce an orexigenic effect when administered via this route has not yet been assessed. The only possible exception is one case study in which a patient suffering from anorexia nervosa was treated intranasally for 1 year with the GHS GHRP-2, and that resulted in an increased feeling of hunger, food intake, and body weight (37). Since ghrelin and GHSs administered peripherally increase subjective feelings of hunger (22, 26, 38), there appears to be an appetite for developing this route of administration and preventing ghrelin degradation using liposome carriers (39, 40). This would have potential use, not only for restoring food intake (eg, in frail older individuals, in those suffering from cachexia or anorexia nervosa) but also to stimulate GH release (eg, for certain forms of GH deficiency and in provocative tests for GH release). To this end, we first selected GHSR agonists and dose based on the ability to induce an orexigenic response in mice after intranasal administration. We then explored, in more detail, its effects on mice meal patterns, Fos protein expression in the arcuate nucleus, including the neurochemical identity of these activated neurons by RNAscope, and also GH release.

## Materials and Methods

### Animals

All experiments were carried out on adult male C57B6/6J mice (Charles River Laboratories), except for the GH measurements, which were carried out on adult female C57B6/6J mice (Charles River Laboratories) due to a less distributed variance in GH pulse amplitude in females compared to males (41-43). Given our aim of demonstrating engagement of the brain ghrelin signaling system by intranasal delivery of GHSR agonists, studies exploring food intake and Fos expression, including neurochemical identification, were limited to male mice; most prior work has used males to validate such effects, presumably linked to the negative effect of estrogen on food intake (as seen in the follicular and preovulatory phase) (44), including that induced by ghrelin (45). Mice of the same sex were group housed (2-8 mice per cage) up to 1 week before the first experimental procedures, after which they were single-housed and left undisturbed for 1 week. Mice were kept at 20 to 22 °C and 50% humidity on a 12-hour dark-light cycle (lights on at 7 Am). Unless otherwise stated, mice had ad libitum access to water and standard chow (2016 Teklad diet; Envigo; 3.0 kcal/g). All experiments were approved by the local Ethics Committee for Animal Care in Gothenburg, Sweden (Göteborgs djurförsöksetiska nämnd; permit No. 2021-3400, approved December 6, 2021) and followed European guidelines (Decree 86/609/EEC).

### Selection of Growth Hormone Secretagogue Receptor Agonist for Subsequent Intranasal Testing

**Figure 1.**
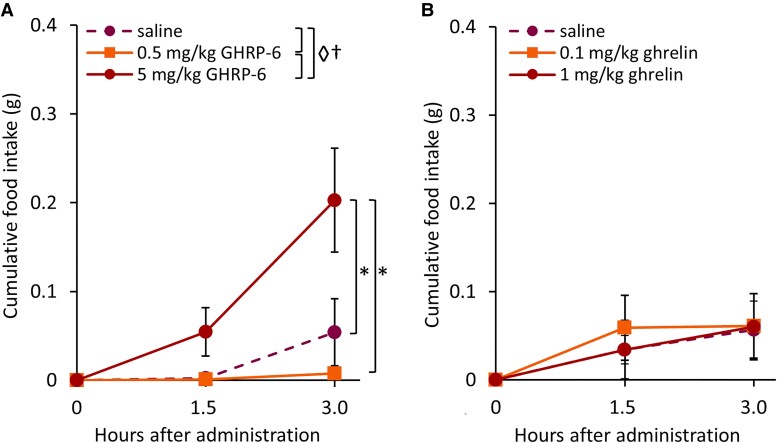
The effects of intranasal delivery of saline or one of 2 GHSR agonists at 2 different doses on cumulative food intake. A, A dose of 5 mg/kg but not 0.5 mg/kg GHRP-6 increased food intake; measurements were taken at baseline, at 1.5 hours and 3.0 hours (n = 6). Two-way analysis of variance (ANOVA) indicated that there was a time effect, a treatment effect, and a time × treatment effect. Tukey post hoc further indicated that food intake after the 5-mg/kg GHRP-6 treatment was significantly increased after 3 hours compared to the saline control and 0.5 mg/kg GHRP-6 dose. B, Intranasal ghrelin did not change cumulative food intake (n = 6). Two-way ANOVA did not indicate an effect of treatment or time. Data are shown as mean ± SEM. Symbols represent: ◊*P* less than .05 (general effect of treatment); †*P* less than .05 (general effect of time); *P less than .05.

In a pilot study, we sought to determine if GHSR agonists, administered via the intranasal route, affect the amount of food eaten in mice (n = 6). We tested ghrelin, GHRP-6, and MK-0677, since all have been shown to increase food intake when administered peripherally (22, 23, 46-49). Mice were gradually habituated to handling and to intranasal administration over 21 days, as described previously (50). Mice received intranasal delivery of saline, ghrelin (No. 1465; Tocris), GHRP-6 (No. G4535, Sigma-Aldrich), or MK-0677 (No. SML0993, Sigma-Aldrich). All GHSR agonists were diluted in a saline vehicle. We tested 2 doses of ghrelin (0.1 and 1 mg/kg), 2 doses of GHRP-6 (0.5 and 5 mg/kg), and 3 doses of MK-0677 (3, 10, and 30 mg/kg). Since in humans GHSR agonists were delivered at a 30-fold higher dose intranasally than intravenously (with GH release as an end point), the upper doses selected here for intranasal delivery to mice were also in the range of at least 30-fold higher than previously used for peripheral administration in rodents (14, 24, 48, 49, 51, 52). No more than 20 µL of the solution (10 µL/nostril) was administered to awake, hand-restrained mice in a horizontal, upside-down position. A micropipette was used to administer the compounds in approximately 5-µL drops at 1-minute intervals, with a 5-minute interval between nostrils. For crossover experiments, mice were allowed 1 washout day between treatments. Food intake was measured manually at 1.5 hours and 3 hours following intranasal administration. When administered by the intranasal route, GHRP-6 at the 5-mg/kg dose (Fig. 1) and MK-0677 (at the 30 mg/kg dose), but not by any of the used doses of ghrelin, induced a feeding response. Mice treated intranasally with MK-0677 exhibited signs of intranasal irritation and had profound expression of Fos protein in the paraventricular nucleus of the hypothalamus (an area activated by stress exposure), neither of which were observed after GHRP-6 or ghrelin delivery. Thus, data from MK-0677–treated mice could not be included in the analysis and further studies with this GHS were not pursued. We were, however, able to select the 5-mg/kg dose of GHRP-6 for further investigation.

### Intranasal GHRP-6 Delivery: Effect on Food Intake and Meal Patterns

Food intake and meal patterns were assessed using the in-cage Feeding Experimentation Device 3 (FED3) (Open Ephys Production Site), enabling uninterrupted and undisturbed recording in a familiar environment (53). This device dispenses a 20-mg chow food pellet each time a pellet is removed, thereby providing a continuous supply of food that is time-stamped. Regular chow was removed when the FED3 was placed in the cage, making dispensed pellets the only available food source. Mice learned to feed from the FED3 over a period of 48 hours, after which the FED3 device was removed, and they were offered regular chow for 1 day prior to the experiment.

The day of the experiment, regular chow was removed from the home cages for 2 hours prior to intranasal administration. Mice received either saline or 5-mg/kg GHRP-6 intranasally as described earlier (n = 7) and returned to the home cage with FED3 devices taking automated food intake measurements for 6 hours. For crossover experiments, mice were allowed 1 washout day between treatments. From the pellet time-stamp data, we performed food pattern analysis using a custom python graphical user interface (Python Software Foundation. Python Language Reference, version 3.12, http://www.python.org). One meal was defined as a minimum of 2 pellets (40 mg) eaten with an interval of less than 5 minutes (54). Food intake was calculated from dispensed pellets per hour, enabling meal size and meal frequency (meals/hour) analysis.

### Neuronal Activation in the Arcuate Nucleus On Intranasal Treatment of GHRP-6

Since GHRP-6 has previously been shown to robustly increase the number of neurons detected that express Fos protein (a neuronal activation marker) in the arcuate nucleus in ad libitum–fed rats after intravenous (14) or intracerebroventricular (55) delivery, in the present study we sought to determine whether this also occurs in mice receiving GHRP-6 by the intranasal route. Mice were fed ad libitum until 2 hours before intranasal delivery, to align their feeding status. Mice received intranasal delivery of either GHRP-6 (5 mg/kg; n = 7) or an equal volume of saline (n = 6). Ninety minutes later, they were deeply anesthetized with a mixture of Ketalar (75 mg/kg; Pfizer AB) and Sedastart vet (1 mg/kg; Produlab Pharma B.V.). Mice were then transcardially perfused with freshly prepared heparinized 0.9% saline followed by 4% paraformaldehyde (PFA) in a 0.1 M phosphate buffer (PB). Brains were dissected and kept in a postfix solution of 4% PFA at 4 °C for 24 hours followed by cryoprotection in 0.1-M autoclaved PB saline containing 25% sucrose. Brains were promptly frozen on dry ice and kept at −80 °C until cryosection. Whole-brain coronal sections (20 µm) were obtained using a cryostat and stored in tissue storage solution at −20 °C until further processing (25% glycerin, 25% ethylene glycol, 50% sterile 0.1-M PB).

Whole-brain, free-floating sections were processed for the immunohistochemical detection of Fos protein using the 3, 3′-diaminobenzidine (DAB)-hydrogen peroxidase method as described previously (56). Following deactivation of endogenous peroxidases, sections were rinsed with 0.1-M PB + 0.3% Triton X-100 and blocked for 1 hour at room temperature in 0.1-M PB, 3% normal goat serum, 0.25% bovine serum albumin, and 0.3% Triton X-100. Sections were then incubated with an anti-c-Fos rabbit primary antibody (dilution 1:1000; No. 226003, RRID: AB_2231974, Synaptic Systems) overnight at room temperature. The following day, sections were rinsed and subsequently incubated for 2 hours with a peroxidase goat-anti-rabbit immunoglobulin (Ig)G secondary antibody (dilution 1:1000; No. A-11032, RRID: AB_2534091, Thermo Fisher Scientific) followed by incubation with a diaminobenzidine, nickel, and hydrogen peroxide solution. Brain sections were mounted onto glass slides and coverslipped with Pertex (Histolab). Unilateral images (2-7 sections per mouse from 1.43-2.03 caudal to bregma) were acquired using a Leica DMRB fluorescence microscope (10X/N.A. 0.30; Leica Microsystems). The number of Fos-positive neurons per hemisection was counted manually and calculated as the average from 3 blind countings, averaged for each brain and, ultimately, averaged for each experimental group.

### RNAscope

We sought to explore the neurochemical identity of arcuate neurons that express *c-fos* mRNA after intranasal delivery of GHRP-6. Sections were processed from the same mice as those for the immunohistochemistry (discussed earlier) and hence, perfused at 90 minutes following intranasal delivery; at this time point, *c-fos* mRNA is expected to remain sufficiently high (57-60). Triple fluorescent in situ hybridization using RNAscope (61) was performed to explore colocalization of mRNAs for *c-fos* (the gene coding for Fos protein), *Ghsr*, *Ghrh*, and *Agrp* in the arcuate nucleus of mice that received a 5-mg/kg dose GHRP-6 intranasally. We did not perform this analysis in the saline-treated group since there were very few Fos-activated neurons in this region and also because we did not expect engagement of orexigenic AgRP or GHRH neurons in this group (15).

All reagents were purchased from Advanced Cell Diagnostics (ACD), unless stated otherwise. The *c-fos* probe (No. 403591-C3) contained 20 oligonucleotide pairs and targeted region 473-1497 (Acc. No. NM_022197.2) of the *c-fos* transcript. The *Ghsr* probe (No. 480031-C1) contained 14 oligonucleotide pairs and targeted region 2 to 742 (Acc. No. NM_032075.3) of the *Ghsr* transcript. The *Agrp* probe (No. 316171-C2) contained 13 oligonucleotide pairs and targeted region 14 to 613 (Acc. No. NM_033650.1) of the *Agrp* transcript. The *Ghrh* probe (No. 470991-C2) contained 11 oligonucleotide pairs and targeted region 2 to 483 (Acc. No. NM_010285.2) of the *Ghrh* transcript. Negative and positive control probes were processed in parallel with the target probes to ensure RNA integrity and an optimal assay performance. Sections of 20 µm were mounted onto SuperFrost Plus slides (No. 631-9483; VWR) and baked at 60 °C overnight in a HybEz oven (No. 321462). The day of the assay, slides were first incubated in 4% PFA for 15 minutes at 4 °C. After washing in demineralized water, the RNAscope protocol was followed as described previously (61). During 2 assays of RNAscope stainings, the *Ghsr*, *Agrp*, and *c-fos* mRNA probes were labeled with Opal 570 (1:2000, No. FP1488001KT, Akoya Biosciences), Opal 520 (1:500, No. FP1487001KT, Akoya Biosciences), and Opal 650 (1:2000, No. FP1496001KT, Akoya Biosciences), respectively, while in the second assay *c-fos*, *Ghrh*, and *Ghsr* mRNA probes were labeled with the same Opal 570, Opal 520, and Opal 650, respectively.

For quantification of the RNAscope data, images were captured using a laser scanning confocal microscope (LSM700 inverted, Zeiss) equipped with a Plan-Apochromat 40/1.3 oil DIC objective (used at the Centre of Cellular Imaging at Gothenburg University). Tile scans (3 × 3) and Z-stacks (optical section of 90 µm) of the arcuate nucleus–containing sections were captured unilaterally. Z-stack images were processed using the maximum intensity projection function in Zen black software (Zeiss) and neurons were automatically counted using QuPath software (version 0.3.0). Neurons were identified by DAPI (4′,6-diamidino-2-phenylindole) staining (a cell nucleus staining) and defined as being positive for a given peptide mRNA when more than 2 fluorescent dots/cell were detected (58, 62). The quantification of the coexpression per hemisection (1-4 sections per mouse from 1.43-2.03 caudal to bregma) was averaged for each brain and, ultimately, for each experimental group.

### Assessment of Growth Hormone Levels On Intranasal Treatment With GHRP-6

To assess whether the blood serum GH response following intranasal administration corresponds to the known response from central/systemic administration of GHSs (63), another cohort of C57B6/6J mice (n = 10) was exposed to intranasal GHRP-6 (5 mg/kg) or saline in a crossover fashion. Mice were habituated to intranasal treatment as described earlier. Blood samples were collected from the lateral saphenous vein from awake mice at 10 to 20 minutes following intranasal treatment. Blood samples were clotted for 30 minutes and centrifuged to obtain serum, aliquoted, and stored at −80 °C until usage. GH levels were measured in duplicate using a commercial enzyme-linked immunosorbent assay kit (No. EZRMGH-45K, RRID: AB_2892711, EMD Millipore) following the manufacturer's instructions. Samples were thawed only once.

### Statistical Analysis

The program IBM SPSS statistics 25 (IBM Corp) was used for statistical analysis. Comparisons were carried out by one-way repeated-measures analysis of variance (ANOVA) when assessing feeding response on intranasal GHRP-6 with treatment (GHRP-6, saline) as “between factor” and time (crossover repeated measurement) as “within factor” variables. Additional paired-sample *t* tests were used to follow up significant main effects and/or interactions. We used paired-sample *t* tests for meal pattern analysis and GH levels and one-sided independent-sample *t* tests for cell activation in the arcuate nucleus.

Plots were generated using Excel and express the mean ± SEM. Statistical significance was set at *P* less than or equal to .05, and values of .05 less than *P* less than .1 were considered evidence of statistical trends. Statistical annotations of the main analysis include the *P* value and its corresponding *F* or *t* ratio together with the degrees of freedom.

## Results

### Selection of Growth Hormone Secretagogue Receptor Agonist and Dose for Subsequent Testing, Using Food Intake as a Primary End Point After Intranasal Administration

Food intake was measured manually for 3 hours following intranasal delivery of 2 doses of GHRP-6 (Fig. 1A) and ghrelin (Fig. 1B) (n = 6, in crossover fashion) based on peripheral administered doses known to elicit a food intake response (19, 38). Repeated-measures 2-way ANOVA showed a general effect of GHRP-6 treatment (F_[2,4]_ = 6.911; *P* = .050), time (F_[1,5]_ = 15.338; *P* = .011), and a time × treatment interaction (F_[2,4]_ = 7.149; *P* = .048) on food intake. Specifically, the 5-mg/kg dose increased food intake compared to saline and 0.5-mg/kg treatment at the 3-hour time point (saline vs 5 mg/kg; *P* = .048; .5 vs 5 mg/kg; *P =* .01). Intranasal ghrelin, at the doses tested, did not increase food intake significantly as we did not find a general effect of ghrelin treatment (F_[2,4]_ = 0.045; *P* = .965) or time (F_[1,5]_ = 3.725; *P* = .112). This led to the selection of the GHSR agonist GHRP-6 at a dose of 5 mg/kg for subsequent experiments.

### Intranasal Administration of GHRP-6 to Mice Increases Food Intake by Increasing the Number of Meals Eaten and Meal Size

**Figure 2.**
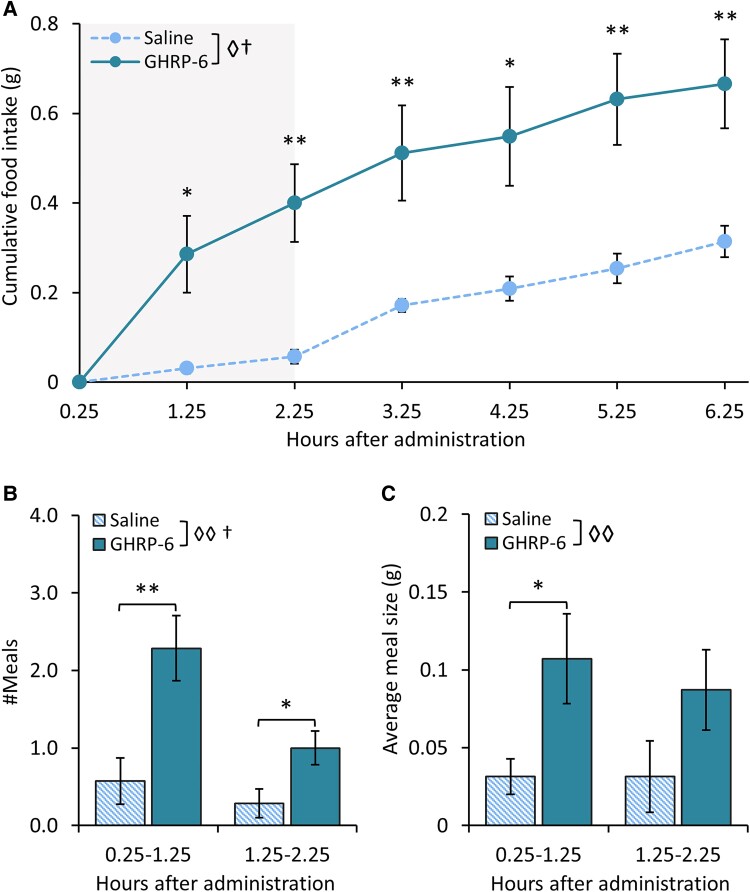
Intranasal GHRP-6 delivery increases food intake by increasing meal frequency and meal size. A, Cumulative chow intake at different time points, starting at 15 minutes after intranasal administration of 5-mg/kg GHRP-6 or saline to mice (n = 7). The gray box marks the time window for analysis of B, meal frequency and C, meal size. Symbols represent: ◊ *P* < .05 (general effect of treatment); †*P* < .05 (general effect of time) *P less than .05; **P less than .01 (5 mg/kg GHRP-6 vs saline). Data are shown as mean ± SEM.

Here we used the FED3 home cage system (n = 7, in crossover fashion) for food intake and meal pattern analysis after 5-mg/mL GHRP-6. Repeated-measures 2-way ANOVA showed a general effect of treatment (F_[2, 6]_ = 11.629; *P* = .014) and time (F_[5, 2]_ = 54.186; *P* = .018) on cumulative food intake, where GHRP-6–treated mice increased their food intake at all time points (1.25 hours; *P* = .019; 2.25 hours; *P* = .002; 3.25 hours; *P* = .008; 4.25 hours; *P* = .011; 5.25 hours; *P* = .004; 6.25 hours; *P* = .006) (Fig. 2A) compared to mice receiving saline intranasally. The most pronounced increase of food intake was observed during the 2-hour window starting 15 minutes after treatment. This was reflected by a general effect of treatment (t_[1,6]_ = 21.146; *P* = .004) and time (t_[1,6]_ = 6.600; *P* = .042), where the number of meals eaten (Fig. 2B) was significantly higher in GHRP-6–treated mice during the first and second hour (1 hour *P* = .006; 2 hours *P* = .028) and a general effect of treatment on average meal size (t_[1,6]_ = 16.251; *P* = .007; Fig. 2C), where we specifically found an increase in GHRP-6–treated mice during the first hour (*P* = .031). Food intake and meal patterns of saline-treated mice were similar to that expected from mice of similar age during the same phase of the light cycle (54).

### Intranasal GHRP-6 Activates Neurons in the Arcuate Nucleus of Mice

**Figure 3.**
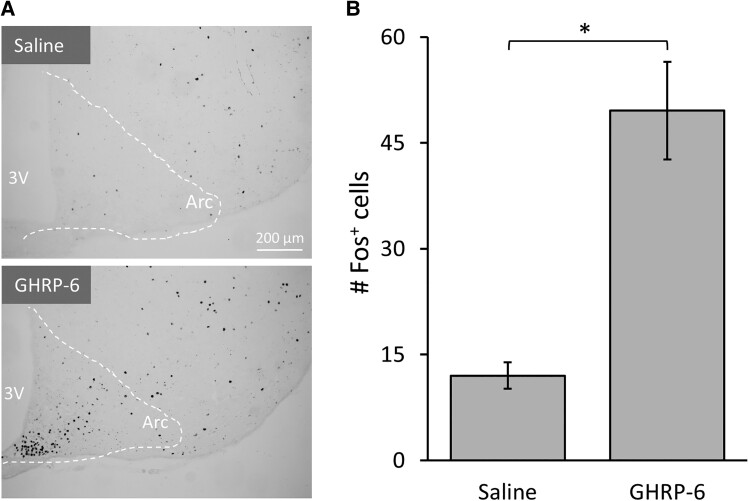
Intranasal GHRP-6 increases the number of Fos expressing neurons in the arcuate nucleus (Arc). A, Representative Fos-DAB images from the Arc showing effects of intranasal GHRP-6 (bregma: −1.67 mm, 20 µm sections). Third ventricle = 3 V. B, Manual unilateral counting of nuclei that express Fos protein from GHRP-6 (n = 7) or saline-treated mice (n = 6). Symbols represent *P less than .05. Data are shown as mean ± SEM.

We delivered 5-mg/kg GHRP (n = 7) or saline (n = 6) intranasally to an additional cohort of mice for visualization of activated (Fos protein–expressing) neurons in the arcuate nucleus, (area shown in Fig. 3A). Consistent with prior studies (14, 55), a one-sided independent-sample *t* test of unilateral counting of Fos-positive neurons showed a statistically significant increase in GHRP-6–treated mice relative to saline controls (t_[11]_ = −4.869; *P* = .0105) (Fig. 3B).

### Neurochemical Identification of Arcuate Nucleus Neurons Activated by Intranasal GHRP-6 Using RNAscope

**Figure 4.**
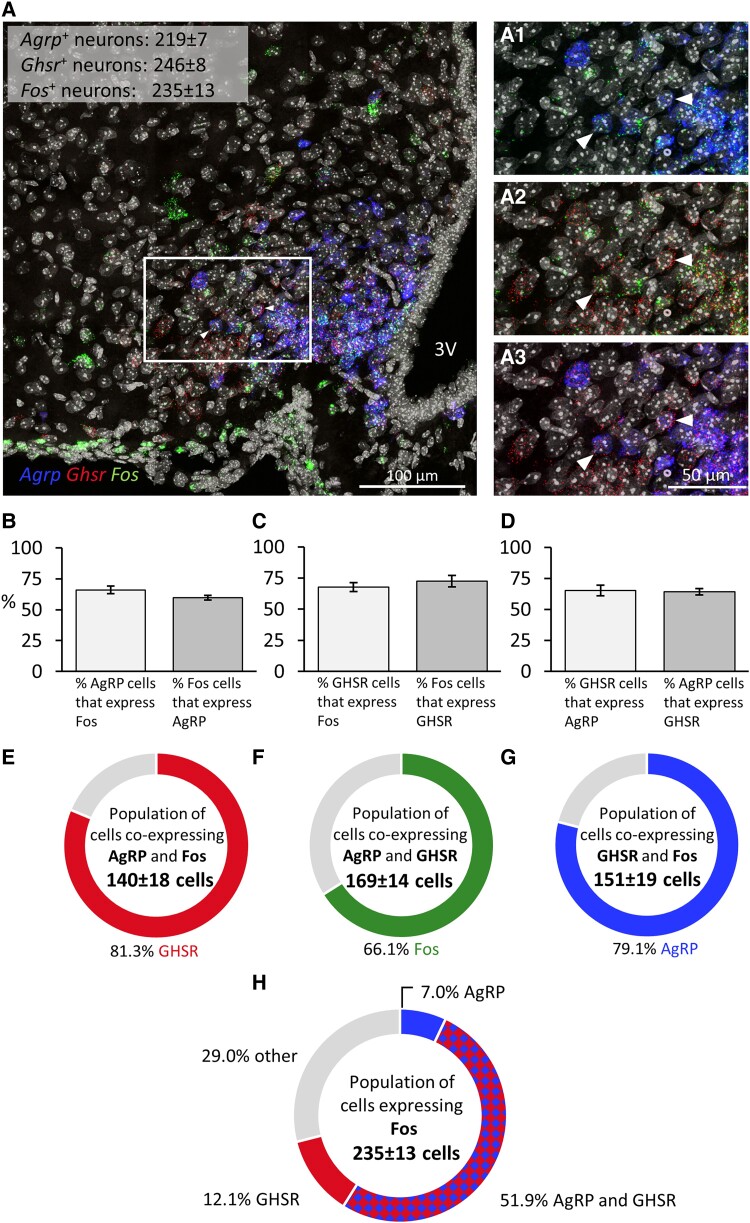
Neurochemical identification of arcuate neurons activated by intranasal GHRP-6 by colocalization of messenger RNAs (mRNAs) for *c-fos*, *Ghsr*, and *Agrp*. A, Representative confocal images of triple RNAscope in situ hypbridization for *c-fos*, *Ghsr*, and *Agrp* in a section showing the arcuate nucleus of a GHRP-6–treated mouse. The panels on the right show the area inside the rectangle, enlarged (A1-A3) with colocalization of *Agrp* and *c-fos* (A1), *Ghsr*, and *c-fos* (A2) and *Agrp* and *Ghsr* (A3). The white arrows in A1 to A3 provide examples of triple-positive neurons. B to D, Bar graphs illustrate colocalization in percentage between mRNA pairs. E to H, Neurochemical identity of E to G, triple-positive neurons and H, *c-fos–*expressing neurons. 3 V = third ventricle, bregma = −1.79. One to 2 hemisections per mouse were quantified (n = 6). Data are shown as mean ± SEM.

**Figure 5.**
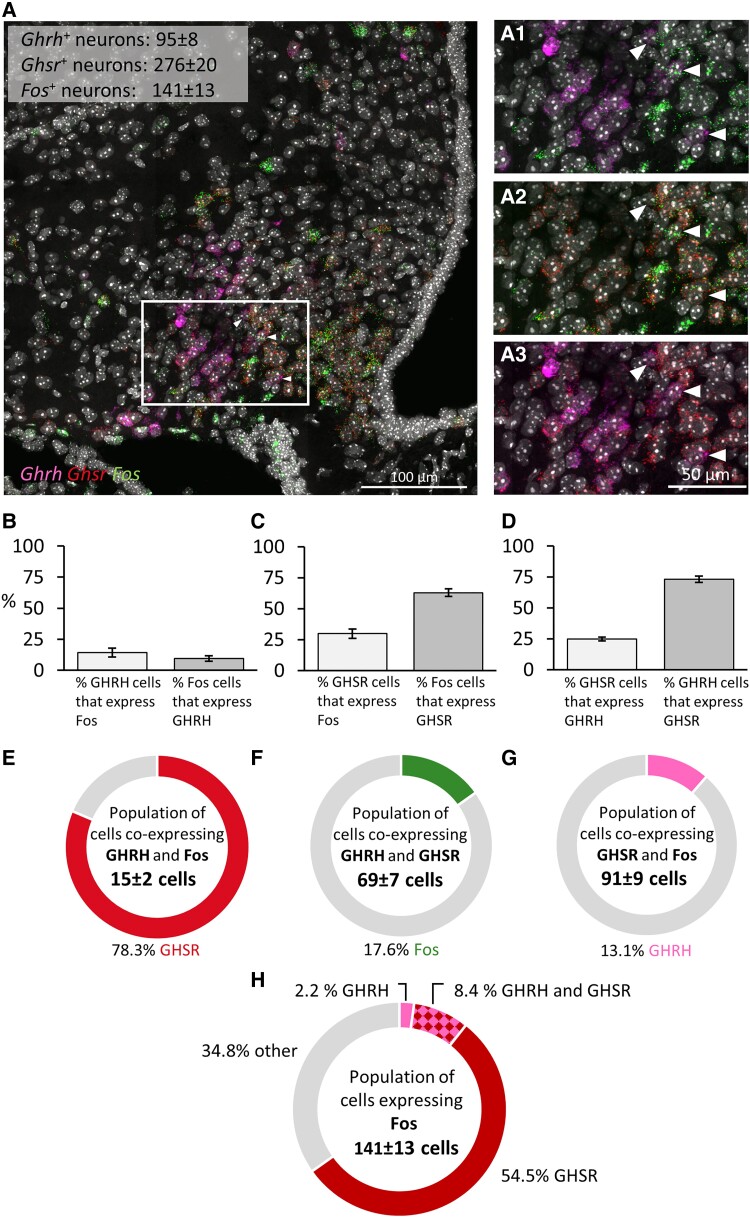
Neurochemical identification of arcuate neurons activated by intranasal GHRP-6 by colocalization of messenger RNAs (mRNAs) for *c-fos*, *Ghsr*, and *Ghrh*. A, Representative confocal images of triple RNAscope in situ hypbridization for *c-fos*, *Ghsr*, and *Ghrh* in a section containing the arcuate nucleus of a GHRP-6–treated mouse. The panels on the right show the area inside the rectangle, enlarged (A1-A3) with colocalization of *Ghrh* and *c-fos* (A1), *Ghsr*, and *c-fos* (A2) and *Ghrh* and *Ghsr* (A3). The white arrows in A1 to A3 give examples of triple-positive neurons. B to D, Bar graphs illustrate colocalization in percentage between mRNA pairs. E to H, Neurochemical identities of E to G, triple-positive neurons and H, *c-fos–*expressing neurons. 3 V = third ventricle, bregma = −1.79. Two to 4 hemisections per mouse were quantified (n = 5). Data are shown as mean ± SEM.

In mice treated intranasally with GHRP-6, we found colocalization in both RNAscope assays, in the first assay for *c-fos*-, *Ghsr*-, and *Agrp* mRNA expression (n = 6; Fig. 4) and in the second assay for *c-fos*-, *Ghsr*-, and *Ghrh* mRNAs (n = 5; Fig. 5). Representative images for each assay illustrate triple colocalization in the arcuate nucleus (see Figs. 4A and 5A), as well as double labeling for each combination of probes (Figs. 4A1-4A3 and 5A1-5A3). Initially, we examined neurons for double labeling (Figs. 4B-4D and 5B-5D). Our results indicate that 58.9 ± 2.1% of *c-fos*–expressing neurons (ie, activated neurons) coexpress *Agrp* (Fig. 4B), while 10.9 ± 1.8% express *Ghrh* (Fig. 5B). Notably, 64.4 ± 1.9% of *c-fos*–expressing neurons also expressed *GHSR*, as averaged from both assays (64.3 ± 2.6%, Fig. 4C, and 64.5 ± 3.0%, Fig. 5C). Additionally, the proportion of *Ghsr*-, *Agrp*-, and *Ghrh*-expressing neurons that also expressed *c-fos* was 65.2 ± 4.2% *Ghsr* in the first assay (see Fig. 4C), 32.9 ± 2.4% *Ghsr* in the second assay (see Fig. 5C), 59.4 ± 5.6% *Agrp* (see Fig. 4B), and 16.5 ± 3.1% *Ghrh* (see Fig. 5B). On the other hand, more than half of the *Ghsr*-expressing neurons (67.7 ± 3.5%) coexpressed *Agrp*. Lastly, 72.4 ± 4.5% of *Agrp*-expressing neurons coexpressed *Ghsr* (see Fig. 4D) and 24.8 ± 1.9% of *Ghsr*-expressing neurons coexpressed *Ghrh* (see Fig. 5D).

Regarding triple coexpressions, we observed that two-thirds of the total population of neurons coexpressing *Agrp* and *Ghsr* (66.1 ± 6.4%) coexpressed *c-fos* (ie, were activated) following intranasal administration of GHRP-6 (Fig. 4E). The majority of the *Agrp*-expressing neurons activated by intranasal GHRP-6 also expressed *Ghsr* (81.3 ± 2.4%; Fig. 4F), and most of the *Ghsr*-expressing neurons activated also expressed *Agrp* (79.0 ± 6.8%; Fig. 4G). Of the total population of neurons coexpressing *Ghrh* and *Ghsr*, 17.6 ± 3.2% were activated following intranasal GHRP-6 administration (Fig. 5F). Moreover, most of the *Ghrh*-expressing neurons activated by intranasal GHRP-6 also expressed *Ghsr* (78.3 ± 2.4%; Fig. 5E); and 13.1 ± 2.3% of the *Ghsr*-expressing neurons activated by intranasal GHRP-6 also expressed *Ghrh* (Fig. 5G). The absolute number of *c-fos*–expressing neurons differed between the two RNAscope assays, likely due to differences in the automated analysis settings used to analyze captured images for the 2 RNAscope studies linked to different levels of background expression (221 vs 141 cells; see Figs. 4C and 5C); however, the percentage of *Ghsr* coexpressing neurons remained consistent (64.3% vs 64.5%; see Figs. 4C and 5C).

Finally, we analyzed the neurochemical identity of *c-fos*–expressing neurons (Figs. 4H and 5H). Among this population, 51.9 ± 2.4% coexpress *Agrp* and *Ghsr*, 12.1 ± 2.8% coexpress *Ghsr* without *Agrp*, and only 7.0 ± 1.4% coexpress *Agrp* without *Ghsr* (see Fig. 4H). Additionally, 29.0 ± 3.1% of the *c-fos*–expressing population expresses neither *Agrp* nor *Ghsr* (see Fig. 4H). Further analysis revealed that, within the *c-fos*–expressing population, 8.4 ± 1.9% coexpress *Ghsr* and *Ghrh*, 2.2 ± 0.4% coexpress *Ghrh*, and 54.5 ± 2.5% coexpress *Ghsr* (Fig. 5H). Notably, 34.8 ± 2.9% of this *c-fos*–expressing population coexpresses neither *Ghsr* nor *Ghrh*.

### Intranasal GHRP-6 Triggers Growth Hormone Release in Mice

**Figure 6.**
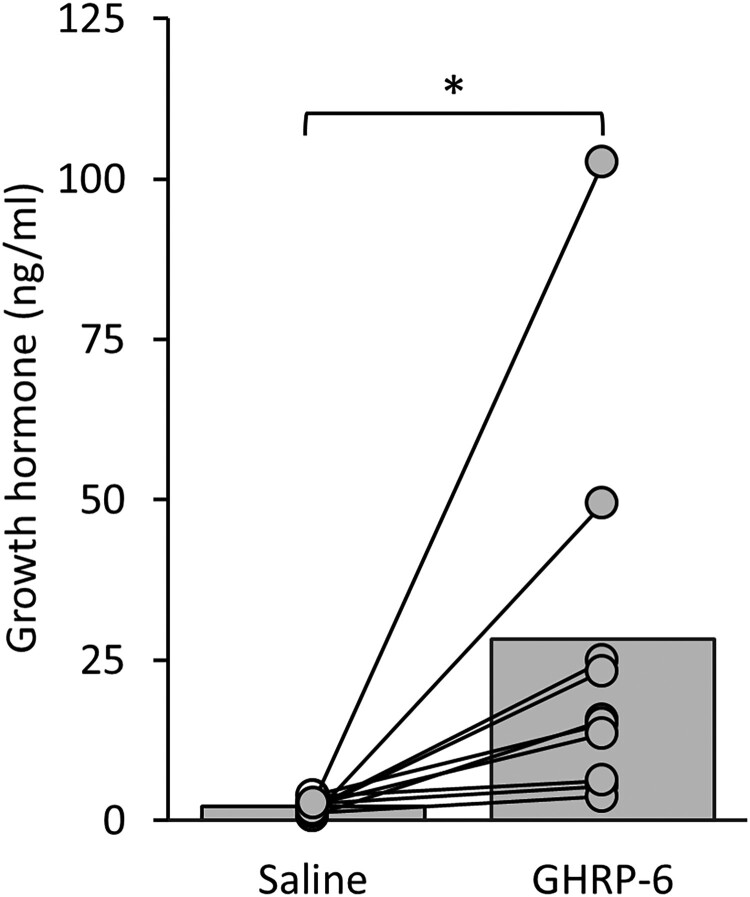
Intranasal delivery of GHRP-6 increases serum growth hormone (GH) levels in mice. GH levels were measured in blood serum samples collected 10 to 20 minutes following intranasal administration of GHRP-6 (5 mg/kg) or saline (n = 10). Symbols represent *P less than .05. Data are shown as mean ± SEM.

At 15 to 20 minutes following intranasal GHRP-6 administration, we observed a statistically significant increase of blood serum GH in the GHRP-6–treated mice compared to saline-treated controls (t_[9]_ = 2.492; *P* = .017; one-sided paired sample *t*-test; Fig. 6).

## Discussion

In the present study, we sought to determine whether the brain ghrelin signaling system can be activated by intranasal delivery of GHSR agonists, reproducing the known effects of these compounds when delivered systemically. Using food intake as a primary screen, we tested 3 different GHSR agonists, namely, ghrelin, the nonpeptide GHS, MK-0677, or the peptide GHS, GHRP-6. Of these, we identified GHRP-6 as the only viable GHSR agonist when administered by this route based on its orexigenic properties, which faithfully reproduce those seen after subcutaneous administration (46), and that it is well-tolerated by mice. This feeding response appears to be due to an increase both in meal size and meal frequency. Consistent with the known target brain systems activated by peripheral or central GHSR agonist delivery (14, 55), we found an increase in the number of neurons detected that express Fos protein in the arcuate nucleus in GHRP-6–treated mice compared to saline-treated controls. We also found an increase of blood serum GH levels after intranasal GHRP-6 delivery, in line with early studies showing such effects after systemic delivery (4). Overall, our data suggest that intranasal GHRP-6 is able to elicit similar effects to those seen after peripheral injection, engaging pathways involved in food intake and GH release.

The ability of intranasal GHRP-6 to increase both meal frequency and meal size translates to mice having more meal initiations, while failing to decrease meal size. The brain pathways involved in meal size and frequency may reside in different brain areas, and it has been suggested that brainstem circuits are sufficient for meal size control while meal frequency is driven by appetitive systems regulating meal initiation in the hypothalamus (64, 65).

For Fos-activation studies, we focused especially on the arcuate nucleus, partly because this is the brain area with the most marked neuronal activation following peripheral delivery of these compounds (14, 15, 60, 66) but also because this is the location of populations involved in both the orexigenic and GH-releasing effects of GHSR agonists. Indeed, systemic GHRP-6 was shown previously to activate approximately 50% NPY neurons and 25% GHRH neurons in this area in rats (15). We found this distribution to be largely comparable to our RNAscope results, where *Agrp*-expressing neurons make up the majority of activated neurons (59.7%); however, the population of activated neurons that expressed *Ghrh* was considerately lower than the 25% reported previously (9.2% vs 25%). The number of neurons coexpressing *Ghsr* with *Ghrh* or *Agrp* agrees with previous studies (67, 68). Our data also highlight the existence of a population of neurons in the arcuate nucleus that are activated by GHRP-6 but do not express GHSR; these may be downstream of GHSR-expressing cells or be activated independently of the GHRP-6 stimulus. Thus, the overall landscape of arcuate nucleus neurons activated by intranasal GHRP-6 aligns with that shown previously for systemic delivery, engaging populations relevant for the orexigenic and GH-releasing effects of GHSR agonists, including ghrelin.

In line with the well-documented GH-releasing properties of GHSs (4, 9, 11), we showed that intranasal GHRP-6 can elicit a potent increase in blood serum GH. We performed this study in female mice since females have a less pulsatile pattern of GH release (42, 43, 69) and anticipated less variable values, as was evident in our saline-treated group. Interestingly, while GHRP-6 delivery increased GH levels in all mice, there was variance regarding the magnitude of this response. Tentatively, this could suggest that in situations where GHSs enhance GH release, pulsatility becomes easier to detect in female mice, in line with studies in males showing that it increases GH pulse amplitude (10, 70). Therefore, it may be that the GH response following intranasal GHRP-6 is amplified but still pulsatile, explaining the greater variability in the GH response to GHRP-6 in our study. Stress (eg, due to acute handling of the mice), which inhibits GH release (71), may be an additional physiological factor that accounts for increased variability in GH response, although we made every effort to reduce this through prior handling and habituation.

To our knowledge, this is the first study to investigate the effects of intranasal ghrelin on orexigenic systems. Based on food intake data testing 2 doses of ghrelin, we did not find evidence that ghrelin can engage orexigenic systems when administered via this route. It may be that we would have seen effects with an even higher dose, but 0.3 mg/kg would certainly induce a feeding response in mice when administered systemically (72). It may be that ghrelin is not transported across the nasal mucosa or is rapidly broken down (eg, by deacetylation), rendering it inactive at the GHSR. GHRP-6 could have the advantage over ghrelin for intranasal administration as it has a lower molecular weight (ghrelin 3311.6, GHRP-6 873.0) and might be easier to be transported across the nasal mucosa (73). Another limitation of intranasal administration of peptides is that they are known to be susceptible to cleavage by nasal enzymes (74). To overcome this, several studies have been developing ghrelin-loaded liposomes (39, 40, 75, 76); however, these studies are still in the early experimental phase. Additionally, as ghrelin remains a costly peptide to produce, especially for the development for human use, intranasal GHRP-6 delivery might prove more cost-effective.

The route by which intranasal GHRP-6 accesses the brain ghrelin signaling system remains unknown. It is possible that it passes into the brain accessing the arcuate nucleus directly, as has been shown for peripheral delivery (77). It may first pass directly into the peripheral circulation to elicit its central nervous system effects. In addition to systemic uptake, two other routes have been proposed for peptides passing from the nose to the brain: one involves internalization by either the olfactory or trigeminal neurons (based on the position of administration in the nose), followed by axonal transport and exocytosis, which takes compounds several hours to reach the brain (73, 78, 79) and has been dismissed as a route for intranasal application of the peptide oxytocin (80). The rapid effect of intranasal GHRP-6, which occurs within the first hour of delivery, may argue against this slow route of access. Intriguingly, olfactory neurons express GHSR (81) and, in the case of ghrelin, uptake into the olfactory bulb from the circulation is rapid (82), although its effects at this site may be more related to meal patterns than food intake per *se* (83). The second route, the paracellular route (between the neurons), is proposed to transport lipophilic and smaller molecules with a cutoff molecular weight of approximately 1000 (84). As the molecular weight of GHRP-6 is 872, this route is more likely, although GHRP-6 is not lipophilic. To elucidate its passage route into the brain would require delivery of labeled GHRP-6, as has been done previously for systemic ghrelin (77, 85), and no such molecule is available currently for GHRP-6.

GHRP-6 has low bioavailability (0.3%) (86). Here, we evidence efficacy after intranasal delivery with relevance for its use in the clinic. We see an opportunity here to provide a noninvasive route for GHSR agonist delivery, and therefore improve adherence, to engage the orexigenic system to support patients with loss of appetite (frail, older individuals, cancer cachexia, etc). In relation to the GH axis, another possible clinical application of intranasal GHRP-6 is as a provocative test in GH-deficient patients (4, 87, 88). It remains unclear which patient groups would benefit most from an enhanced GH response; this requires that pituitary somatotrophs be able to produce GH. There are indications that GHSs could even provide therapeutic benefit by stimulating GH and growth in pediatric patients with GH deficiency (89, 90) and also stimulating GH release in older adults (91). Avoiding daily injections and replacing them with a noninvasive nasal spray holds potential for these patient groups and could improve adherence to GH therapy (92-94).

Our findings suggest that intranasal GHRP-6, but not ghrelin, may engage neurons in the arcuate nucleus involved in food intake, as indicated by increased *c-fos* expression and by the colocalization of these cells with *Ghsr* and *AgRP* mRNAs. Additionally, *c-fos*–expressing cells colocalized *Ghrh* mRNA and intranasal GHRP-6 elevated serum GH levels, indicating engagement of the hypothalamo-pituitary growth axis after intranasal delivery. Further studies are needed to confirm the specific neuronal mechanisms underlying these effects.

## Data Availability

Some or all data sets generated during and/or analyzed during the present study are not publicly available but are available from the corresponding author on reasonable request.

## Abbreviations

AgRP: agouti-related peptide
ANOVA: analysis of variance
DAB: 3, 3′-diaminobenzidine
FED3: Feeding Experimentation Device 3
GH: growth hormone
GHRH: growth hormone–releasing hormone
GHRP-6: growth hormone–releasing peptide-6
GHS: growth hormone secretagogue
GHSR: growth hormone secretagogue receptor
mRNA: messenger RNA
NPY: neuropeptide Y
PB: phosphate buffer
PFA: paraformaldehyde
